# A Fuzzy Virtual Actuator for Automated Guided Vehicles

**DOI:** 10.3390/s20154154

**Published:** 2020-07-26

**Authors:** Ralf Stetter

**Affiliations:** Department of Mechanical Engineering, Ravensburg-Weingarten University (RWU), 88250 Weingarten, Germany; stetter@rwu.de

**Keywords:** virtual sensors, virtual actuators, fault-tolerance, automated guided vehicles

## Abstract

In the last decades, virtual sensors have found increasing attention in the research community. Virtual sensors employ mathematical models and different sources of information such as actuator states or sensors, which are already existing in a system, in order to generate virtual measurements. Additionally, in recent years, the concept of virtual actuators has been proposed by leading researchers. Virtual actuators are parts of a fault-tolerant control strategy and aim to accommodate faults and to achieve a safe operation of a faulty plant. This paper describes a novel concept for a fuzzy virtual actuator applied to an automated guided vehicle (AGV). The application of fuzzy logic rules allows integrating expert knowledge or experimental data into the decision making of the virtual actuator. The AGV under consideration disposes of an innovative steering concept, which leads to considerable advantages in terms of maneuverability, but requires an elaborate control system. The application of the virtual actuator allows the accommodation of several possible faults, such as a slippery surface under one of the drive modules of the AGV.

## 1. Introduction

The flexibility and agility of production systems are of paramount importance because the complexity and diversity of most products are still increasing and production networks are more and more dynamic and internationally connected. Automated guided vehicles (AGVs) are one prominent means for the realization of flexible and agile product systems, because they combine a large degree of reconfigurability with relatively low investment costs. One cornerstone for the successful application of AGVs is appropriate control and diagnosis systems. In the last decades, powerful control and diagnosis systems were developed for this kind of application, but the fault-tolerance of AGV systems remains a challenge. AGVs need to precisely determine the current position, their motion state and specified environmental conditions as well as to act in a controlled manner. As conventional control solutions can lead to unsatisfactory performance or even instability [1], fault-tolerant control systems are proposed, which manage and compensate possible malfunction and faults of sensors, actuators and other internal and external system elements. In this area, virtual sensors and virtual actuators are a promising approach addressing the challenge of fault-tolerance. A current development is fuzzy virtual actuators [2], which combine reliable algorithms with the advantages of fuzzy control such as powerful approximation methods or the inclusion of expert knowledge. This paper proposes a fuzzy virtual actuator for AGVs, which allows combining the information from more than one residual (an identified difference between an analytical model and a sensor reading) and the incorporation of expert knowledge. From this, the central research question can be derived:

How can a control and diagnosis subsystem for an AGV be developed, which realizes a fuzzy virtual actuator that uses the information of residuals created with an appropriate analytical model of the AGV and combines this information with expert knowledge or experimental data in order to increase the fault-tolerance of the AGV.

In order to address this research question, this paper starts with a detailed state of the art (Section 2). The application example—a unique transportation platform—is explained in Section 3. In Section 4, the mathematical model that serves as analytical redundancy and in Section 5, the virtual sensor that detects faults and creates residual information are elucidated. The core of this paper—the design of the fuzzy virtual actuator—is explained in Section 6. An evaluation of this design on the basis of the transportation platform is described in Section 7. The final section contains the conclusions and an outlook on further research activities.

## 2. State of the Art

**Figure 1:**
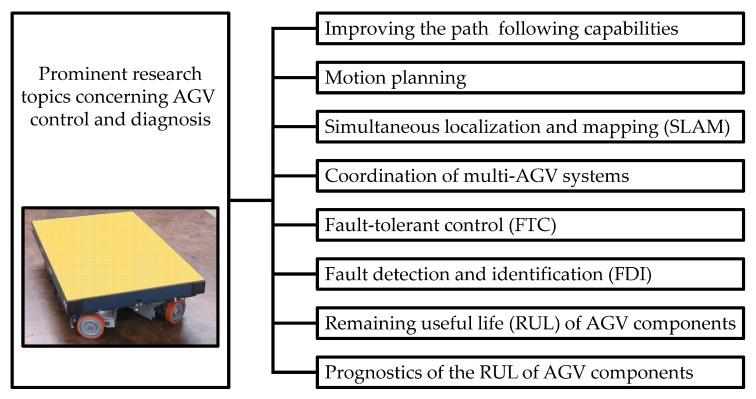
Prominent research topics.

Current research activities concerning AGV control aim at further improving the path following capabilities, for instance a robust H*∞* output-feedback control strategy is refined and applied in [3]. Another central issue in AGV control is the motion planning; several researchers worldwide address this issue [4,5]. Several research groups look into navigation tasks and self-orientation, such as simultaneous localization and mapping (SLAM) [6,7,8,9]. Yet another important topic is the coordination of multi-AGV systems; in this field, the so-called integrated Markov neural reliability computation method [10], cloud solutions [11], colored Petri nets (CPNs) [12], conflict avoidance strategies [13], re-scheduling algorithms [14], dynamic fuzzy cognitive maps [15], as well as fuzzy logic and genetic algorithms [16], are applied. The enormous importance of fault-tolerant control (FTC) as well as fault detection and identification (FDI) is addressed by several research activities. So far, the main points of interest were faults of the steering system [17]; it was, amongst others, proposed to model the AGVs in the form of switching linear parameter varying (LPV) systems [18]. Current research initiatives also try to incorporate the consideration of the remaining useful life (RUL) [19] into AGV control and diagnosis and extend the FDI towards the prognostics of the RUL and the respective confidence intervals [20]. The precedent research topics are summarized in Figure 1.

It can be concluded that fault-tolerance is a central research focus, because commercial products such as cars as well as production systems in industry have become even more complicated and complex over the last decades. This complexity can only be dealt with by employing elaborate control and diagnosis systems [21]. These systems require reliable sensor data, but the possibility to add additional sensors is often limited due to cost, space and weight constraints. One promising approach to tackle this issue is the application of virtual sensors. These sensors generate (virtual) measurements by means of combining sources of information that are already present in a system (such as actuator states and sensors originally intended to provide other measurements) and mathematical models of the system. Several researchers have started to develop and implement virtual sensors, especially in connection with Fault-Tolerant Control (FTC) and Fault Diagnosis and Identification (FDI) [21,22,23,24]. Three different main directions for realizing virtual sensors can be distinguished:

- Observer-based approaches [25,26] (employing, e.g., quadratic boundedness [27]); in some cases based on fuzzy logic [28,29],
- Filter-based approaches (employing, e.g., Kalman filters) [30,31],
- Parameter identification-based approaches [32].

**Figure 2:**
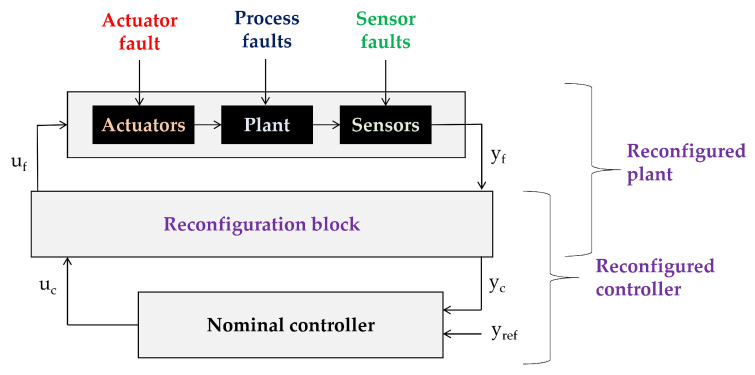
Principle of control reconfiguration with a reconfiguration block.

In recent years the concept of virtual sensors was complemented with the concept of virtual actuators. Initially this concept was proposed by Blanke et al. in the 2003 edition of their book [33]. They use the notion “virtual actuator” for the reconfiguration block in a control reconfiguration scheme (see Figure 2—compare [34]).

This concept could be implemented in several applications. De Oca and Puig [35] propose the use of this kind of virtual actuator for controlling a two-degree of freedom helicopter. Seron et al. describe the application of more than one virtual actuator (a bank of actuators) to the lateral control of an aircraft [36]. The fault-tolerant control of wheeled omnidirectional robots was in the center of the research of Rotondo et al. [18]. General aspects of the application to linear parameter varying systems were later investigated by Rotondo at al. [37,38]; they underline that a virtual actuator is a fault hiding technique that intends to reconfigure the faulty plant instead of the controller. Current works also investigate virtual actuators for the fault-tolerant control of discrete-time descriptor systems [39], adaptive virtual actuators for the fault-tolerant control of linear systems [40] and actuator fault-tolerant control, which is based on probabilistic ultimate bounds [41]. Several research groups investigate the application of fuzzy logic for fault-tolerant control. Ting et al. [42] present a fuzzy reasoning system based on fuzzy reasoning and verification Petri nets (FRVPNs) for fault detection and diagnosis. A fuzzy residual evaluation is proposed by Gentil et al. [43]. Escobet et al. [44] employ a hybrid methodology based on fuzzy and pattern recognition techniques for fault diagnosis. An active sensor fault-tolerant output feedback tracking control is proposed by Shaker and Patton [45]. Vafamand et al. describe a robust controller design for continuous-time Takagi-Sugeno (TS) systems [46]. These research activities lead to an enormous potential to increase the fault-tolerance of technical systems, but do not focus on virtual actuators. First, research works investigate the possibility of fuzzy virtual actuators [2,47]. However, the main focus is on a representation of a nonlinear system employing the Takagi–Sugeno fuzzy model (compare [48]) and not on the application of fuzzy rules for including expert knowledge in the accommodation of faults.

An adjacent field also relying on virtual actuation concerns the vibration control of mechanical systems using, for instance, virtual dynamic vibration absorbers. Using this notion, Wu and Shoa present a control algorithm emulating a dynamic vibration absorber, which allows variable stiffness, inertia and damping coefficients [49]. The application to a flexible arm is described by Bian and Gao [50]. Other research works based on this general approach concern resonant control using a collocated sensor–actuator pair with acceleration, velocity or position feedback [51] and the investigation of virtual dynamic absorbers for vibration control in rotary systems [52]. Further applications concern aero-elastic vibration suppression [53] and mass–spring–damper mechanical systems [54]. In this field of vibration absorption and vibration control, a phase-based fuzzy logic controller is also proposed by Liao et al. [55]. It is important to note that in current industrial settings, AGVs have to be integrated in the so-called Internet of Things (IoT). Several prominent research works investigate approaches for enhancing the fault-tolerance in distributed and wireless environments, for instance, systems based on the non-dominated sorting based genetic algorithm (A-NSGA) [56] or actuator failure avoidance online charging schemes (AFAC) [57]. Currently, approaches to address the challenges resulting from interruptions and noise in decentralized tracking systems are reported [58].

In spite of the successful application of virtual actuators in some precedently listed cases, it has to be pointed out that the plants need to have to general capability to accommodate the respective fault. It is an obvious fact that a fault can only be accommodated if the actuators in the system are (still) capable of achieving a safe and sufficient system state. Current research activities, which intend to achieve a combination of fault-tolerant design and fault-tolerant control [20], have identified the methods over-actuation (compare [59,60]) and actuator overlap as means for holistic fault accommodation. It is therefore proposed to expand the definition of virtual actuators as follows:

**Definition** **1.** *A virtual actuator consists of an appropriate means for control reconfiguration in a system that is able to detect and identify faults and to accommodate these faults through a conscious design of the sensor and actuator structure.*

On the basis of this definition, a holistic application of a virtual actuator is explained in the subsequent sections. Special attention is given to the inclusion of expert knowledge by means of rules formulated in fuzzy logic.

## 3. Application Example: A Transportation Platform

**Figure 3:**
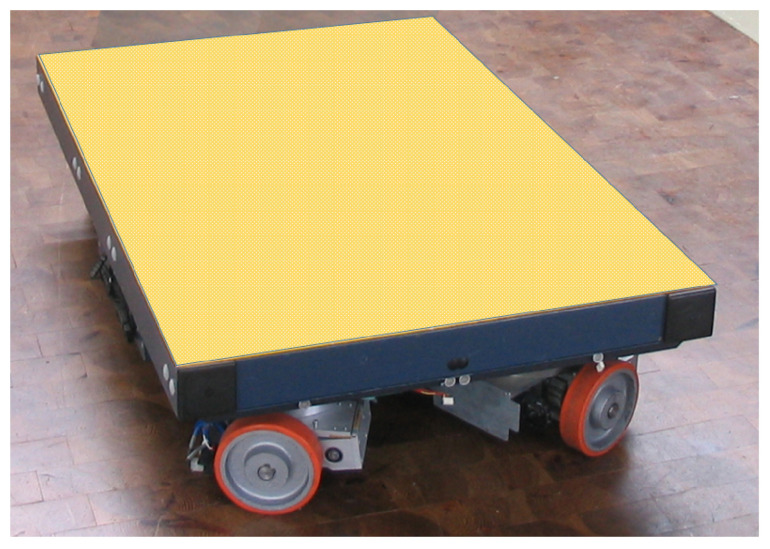
Transportation platform.

AGVs intend to replace static systems such as conveyor belts as well as manual labor. A current development is omnidirectional platforms, which are able to perform complex maneuvers in confined spaces because of their ability to drive in any direction (a prominent example is the KUKA omniMove (KUKA AG, Augsburg Germany)) [6,7]. An omnidirectional production platform with distinct advantages concerning slip behavior and accuracy was developed at the Ravensburg-Weingarten University (RWU) [61]; this platform is shown in Figure 3.

**Figure 4:**
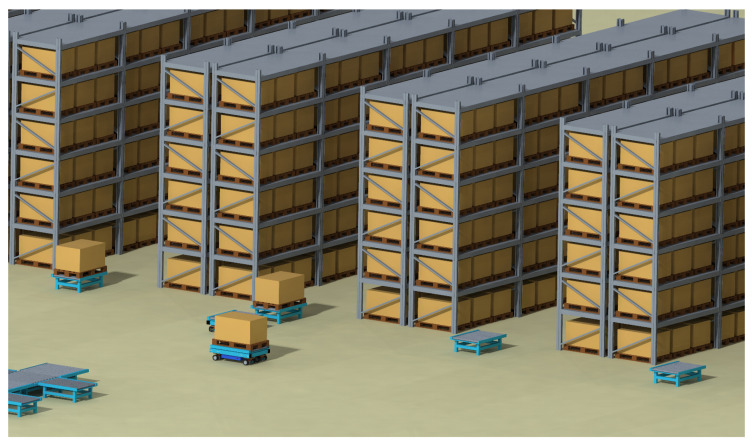
Transportation platform in a high-shelf warehouse.

This platform relies on a unique steering principle, which is based on torque differences between the driven wheel and a modular conception with four identical driving modules, which each consist of two driven wheels. A detailed evaluation of the applicability is described in [62]. An application example of the transportation platform in a high-shelf warehouse is shown in Figure 4. In this application scenario, the transportation platform serves for transporting packaged goods from a production outlet (visible in front) to several transfer stations.

## 4. Mathematical Model of the Transportation Platform

This section explains the mathematical model that enables the control and diagnosis of the transportation platform. The notation is given in Table 1.

It is important to note that this platform allows different modes of driving such as straight driving and Ackermann steering driving (similar to cars; the back wheels remain straight) for high speeds and parallel driving at very low speeds.

**Figure 5:**
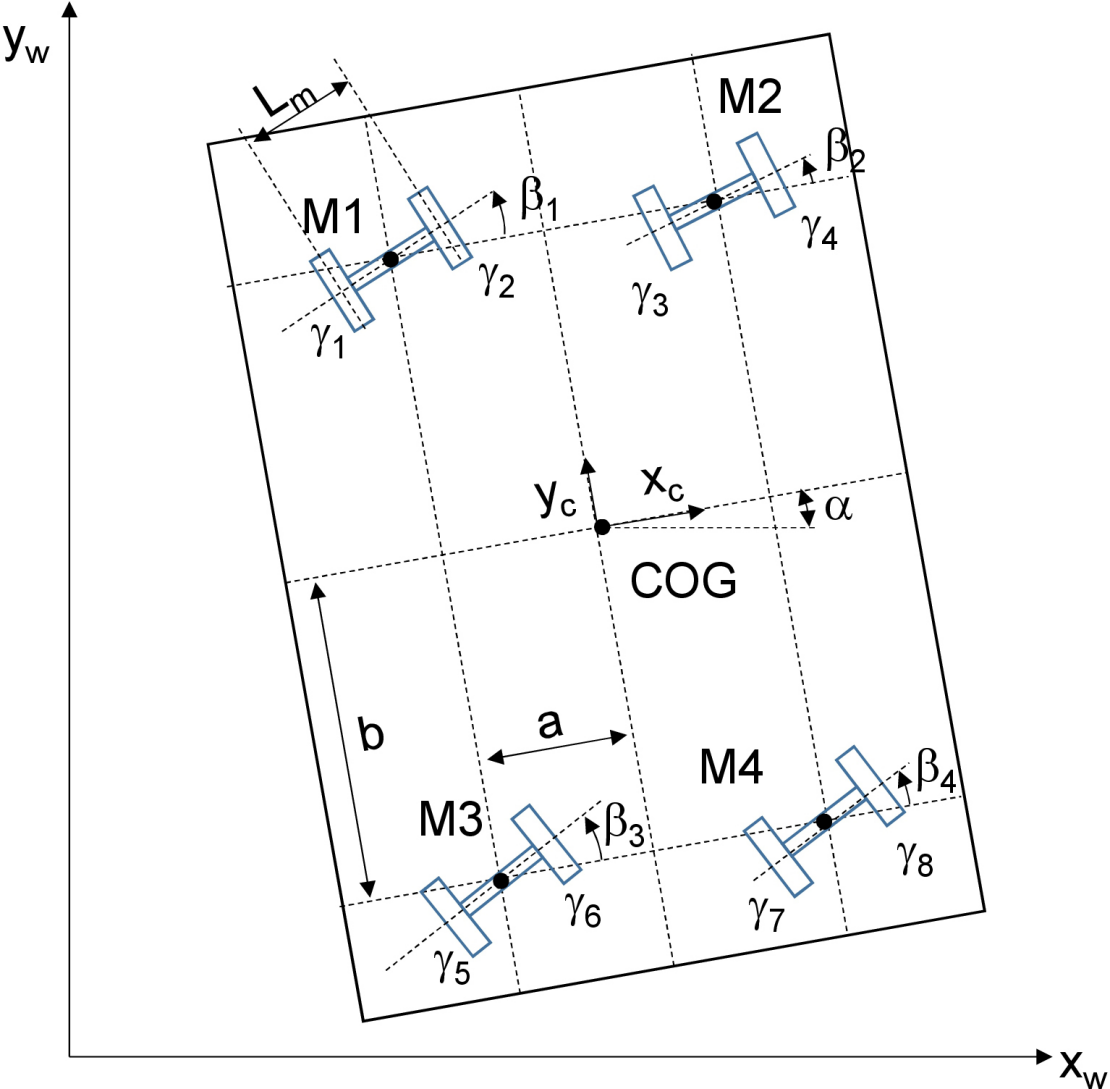
Steering system with kinematic parameters.

The principal steering system of the production platform with the important kinematic parameters is explained in Figure 5.

**Figure 6:**
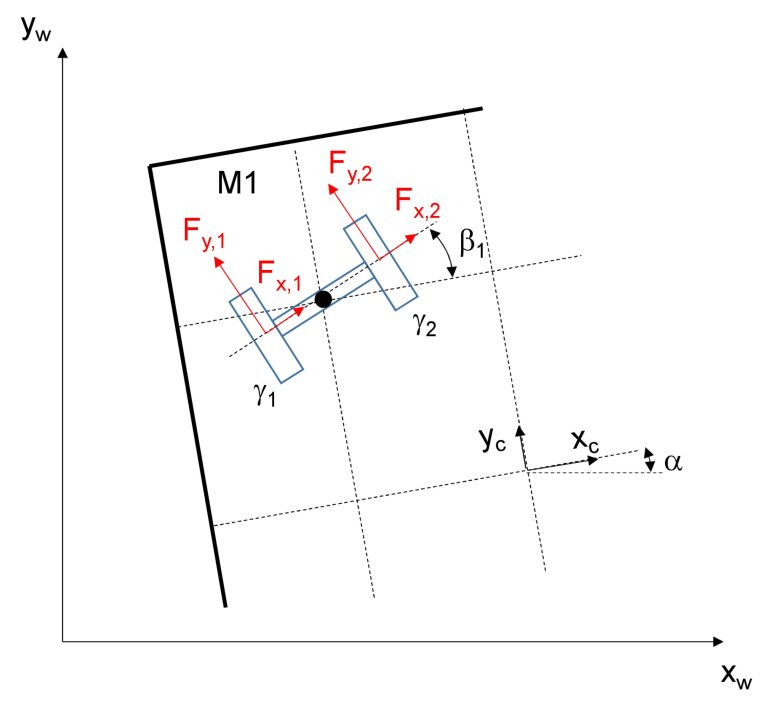
Forces acting on a driving module.

The main acting forces of one module are shown in Figure 6.

The essential parameters of the production platform are given in Table 2.

Analyzing the forces acting on the whole vehicle, it can be derived that the force leading to a longitudinal motion can be found by the subsequent equation:

(1) $$\begin{array}{cc} F_{yc} & =\sum_{j=1}^{2}\cos\left(\beta_{1}\right)F_{y,j}+\sum_{j=3}^{4}\cos\left(\beta_{2}\right)F_{y,j}+\sum_{j=5}^{6}\cos\left(\beta_{3}\right)F_{y,j}+\sum_{j=7}^{8}\cos\left(\beta_{4}\right)F_{y,j}\\ \;\; & +\sum_{j=1}^{2}\sin\left(\beta_{1}\right)F_{x,j}+\sum_{j=3}^{4}\sin\left(\beta_{2}\right)F_{x,j}+\sum_{j=5}^{6}\sin\left(\beta_{3}\right)F_{x,j}+\sum_{j=7}^{8}\sin\left(\beta_{4}\right)F_{x,j}. \end{array}$$

The lateral forces can be derived using a similar equation:

(2) $$\begin{array}{cc} F_{xc} & =\sum_{j=1}^{2}-\sin\left(\beta_{1}\right)F_{y,j}+\sum_{j=3}^{4}-\sin\left(\beta_{2}\right)F_{y,j}+\sum_{j=5}^{6}-\sin\left(\beta_{3}\right)F_{y,j}+\sum_{j=7}^{8}-\sin\left(\beta_{4}\right)F_{y,j}\\ \;\; & +\sum_{j=1}^{2}\cos\left(\beta_{1}\right)F_{x,j}+\sum_{j=3}^{4}\cos\left(\beta_{2}\right)F_{x,j}+\sum_{j=5}^{6}\cos\left(\beta_{3}\right)F_{x,j}+\sum_{j=7}^{8}\cos\left(\beta_{4}\right)F_{x,j}. \end{array}$$

A consideration of the dynamic behavior allows the subsequent formulations:

(3) $${\ddot{y}}_{c}\cdot m=F_{yc},$$

(4) $${\ddot{x}}_{c}\cdot m=F_{xc}.$$

The longitudinal wheel forces obey the subsequent equation:

(5) $$I_{w}\ddot{\gamma_{j}}=p_{j}T-F_{y,j}R_{e}.$$

In this equation, the torque distribution coefficients $p_{j}$ are assumed to be known. Using this information, the required torque for each drive motor can also be calculated:

(6) $$T_{j}=R_{e}\cdot p_{j}\cdot \frac{\kappa \cdot m\cdot \ddot{y_{c}}+m\cdot g\cdot \mu_{r}}{cos\beta_{1}}\;for\;j=1,2,$$

(7) $$T_{j}=R_{e}\cdot p_{j}\cdot \frac{\kappa \cdot m\cdot \ddot{y_{c}}+m\cdot g\cdot \mu_{r}}{cos\beta_{2}}\;for\;j=3,4,$$

(8) $$T_{j}=R_{e}\cdot p_{j}\cdot \frac{\kappa \cdot m\cdot \ddot{y_{c}}+m\cdot g\cdot \mu_{r}}{cos\beta_{3}}\;for\;j=5,6,$$

(9) $$T_{j}=R_{e}\cdot p_{j}\cdot \frac{\kappa \cdot m\cdot \ddot{y_{c}}+m\cdot g\cdot \mu_{r}}{cos\beta_{4}}\;for\;j=7,8.$$

It is also possible to derive the yaw rate dynamics using the subsequent equation:

$$\begin{array}{c} I_{z}\cdot \ddot{\alpha }=-a\cdot \left(\cos\beta_{1}\cdot F_{y,1}+\sin\beta_{1}\cdot F_{x,1}+\cos\beta_{1}\cdot F_{y,2}+\sin\beta_{1}\cdot F_{x,2}\right)\\ +b\cdot (\sin\beta_{1}\cdot F_{y,1}-\cos\beta_{1}\cdot F_{x,1}+\sin\beta_{1}\cdot F_{y,2}-\cos\beta_{1}\cdot F_{x,2})\\ +a\cdot (\cos\beta_{2}\cdot F_{y,3}+\sin\beta_{2}\cdot F_{x,3}+\cos\beta_{2}\cdot F_{y,4}+\sin\beta_{2}\cdot F_{x,4})\\ +b\cdot (\sin\beta_{2}\cdot F_{y,3}-\cos\beta_{2}\cdot F_{x,3}+\sin\beta_{2}\cdot F_{y,4}-\cos\beta_{2}\cdot F_{x,4})\\ -a\cdot (\cos\beta_{3}\cdot F_{y,5}+\sin\beta_{3}\cdot F_{x,5}+\cos\beta_{3}\cdot F_{y,6}+\sin\beta_{3}\cdot F_{x,6})\\ -b\cdot (\sin\beta_{3}\cdot F_{y,5}-\cos\beta_{3}\cdot F_{x,5}+\sin\beta_{3}\cdot F_{y,6}-\cos\beta_{3}\cdot F_{x,6})\\ +a\cdot (\cos\beta_{4}\cdot F_{y,7}+\sin\beta_{4}\cdot F_{x,7}+\cos\beta_{4}\cdot F_{y,8}+\sin\beta_{4}\cdot F_{x,8})\\ -b\cdot (\sin\beta_{4}\cdot F_{y,7}-\cos\beta_{4}\cdot F_{x,7}+\sin\beta_{4}\cdot F_{y,8}-\cos\beta_{4}\cdot F_{x,8}). \end{array}$$

Additionally, the yaw rate dynamics can be evaluated for each of the drive modules:

(10) $$I_{m}\cdot \ddot{\beta_{1}}=-F_{y,1}\cdot \frac{L_{m}}{2}+F_{y,2}\cdot \frac{L_{m}}{2},$$

(11) $$I_{m}\cdot \ddot{\beta_{2}}=-F_{y,3}\cdot \frac{L_{m}}{2}+F_{y,4}\cdot \frac{L_{m}}{2},$$

(12) $$I_{m}\cdot \ddot{\beta_{3}}=-F_{y,5}\cdot \frac{L_{m}}{2}+F_{y,6}\cdot \frac{L_{m}}{2},$$

(13) $$I_{m}\cdot \ddot{\beta_{4}}=-F_{y,7}\cdot \frac{L_{m}}{2}+F_{y,8}\cdot \frac{L_{m}}{2}.$$

It is assumed that the measurement vector

(14) $$\begin{array}{c} y={\left[\dot{\alpha },\dot{\beta_{1}},\cdots ,\dot{\beta_{4}},\dot{\gamma_{1}},\cdots \dot{\gamma_{8}}\right]}^{T} \end{array}$$

is available. The state vector is analogously:

(15) $$\begin{array}{c} x={\left[\dot{\alpha },\dot{\beta_{1}},\cdots ,\dot{\beta_{4}},\dot{\gamma_{1}},\cdots \dot{\gamma_{8}}\right]}^{T}. \end{array}$$

From the precedent elaborations, a state space model can be developed, which will allow a model predictive control of the orientation of the drive modules, the fault diagnosis of certain kinds of faults (reduced friction, reduced torque) and a fault tolerant control based on the virtual actuator concept. The state space model can be given as:

(16) $$\begin{array}{c} G\left(\beta_{i}\right)\dot{x_{}}=B\left(\beta_{i}\right)u_{}+E\left(\beta_{i}\right)d_{}. \end{array}$$

In this equation *x* denotes the state, *u* denotes the control input and *d* denotes the unknown input. The control input is:

(17) $$\begin{array}{c} u_{}={\left[{\ddot{x}}_{c},{\ddot{y}}_{c}\right]}^{T}. \end{array}$$

The unknown input, which is to be estimated, is:

(18) $$\begin{array}{c} d_{}={\left[F_{y,1},F_{y,2},F_{y,3},F_{y,4},F_{y,5},F_{y,6},F_{y,7},F_{y,8},T\right]}^{T}. \end{array}$$

The system matrices in this state space model are described in Appendix A. This system can also be discretized using the Euler methods:

(19) $$\begin{array}{c} G_{k}x_{k+1}=G_{k}x_{k}+B_{k}u_{k}+E_{k}d_{k}+Ww_{k}, \end{array}$$

with

(20) $$\begin{array}{cc} G_{k} & =G\left(\beta_{i}\right),\;B_{k}=T_{s}\cdot B\left(\beta_{i}\right), \end{array}$$

(21) $$\begin{array}{cc} \;E_{k} & =T_{s}\cdot E\left(\beta_{i}\right), \end{array}$$

where $w_{k}$ stands for an exogenous disturbance vector (which includes the discretization error) with a known distribution matrix W. $G(\beta_{i})$, $B(\beta_{i})$ and $E(\beta_{i})$ are obtained by substituting the steering angles of the modules into the matrices of the state space model.

## 5. Fault Detection and Identification with a Virtual Sensor

The initial step for the application of a virtual actuator is the detection of possible faults. It is important to note that the matrix $G_{k}$ can be either non-singular or singular. This leads to the fact that the system described in Equation (19) can be either a linear time-varying system or a descriptor time-varying linear system. It is also important to note that the system (19) has an unknown input $d_{k}$, which has to be estimated in order to allow fault detection and identification. In prior work, a novel adaptive estimator was proposed for a similar task [27]. This estimator is able to estimate the unknown input $d_{k}$ without an estimation of the system state $x_{k}$ and can handle the general issue of the time-varying matrix $G_{k}$ of the system. For the given vehicle, another possibility to address this problem is based on a detection of whether the system is a descriptor time-varying linear system or close to a descriptor time-varying linear system, and which condition may also lead to numerical difficulties and imprecision. In the given case, the system will be a descriptor time-varying linear system if and if only the parameter $p_{a}$ will be zero. Consequently, it can be tested if this parameter is lower than a defined threshold by using the following decision structure:

(22) $$\begin{array}{c} \left\{\begin{array}{c} if\;|p_{a}\left|<\right|T_{d}|\;then\;descriptor\;system\\ else\;linear\;system \end{array}\right. \end{array}$$

where $T_{d}$ denotes a descriptor threshold which is set by the control engineers depending on the numerical capabilities of the control system and the noise level. The rest of this paper will concentrate on the case that the absolute value of the parameter $p_{a}$ is smaller than the descriptor threshold and that a descriptor time-varying linear system is present. For the given vehicle this case can be described by a reduced system description taking into consideration physical relationships:

(23) $$\begin{array}{c} G_{r,k}x_{r,k+1}=G_{r,k}x_{r,k}+E_{r,k}d_{k}+W_{r}w_{r,k}, \end{array}$$

with matrices $G_{r,k}$ and $E_{r,k}$ derived from matrices $G_{k}$ and $E_{k}$, by eliminating the elements that concern the state element $\dot{\alpha }$. The corresponding measurement can be described with the equation:

(24) $$\begin{array}{c} y_{r,k}=C_{r,k}x_{r,k}+Vv_{k}, \end{array}$$

where $v_{k}$ denotes the measurement noise with a known distribution matrix V. It is now possible to apply an unknown input estimator similar to the one proposed by Gillijns and De Moor [63]. This is based on a recursive filter, which has in this case the following form:

(25) $$\begin{array}{c} {\hat{x}}_{k|k-1}=G_{r,k-1}{\hat{x}}_{k-1|k-1}, \end{array}$$

(26) $$\begin{array}{c} {\hat{d}}_{k-1}=M_{k}\left(y_{k}-C_{r,k}{\hat{x}}_{k|k-1}\right), \end{array}$$

(27) $$\begin{array}{c} {\hat{x}}_{k|k}^{*}={\hat{x}}_{k|k-1}+G_{r,k-1}{\hat{d}}_{k-1}), \end{array}$$

(28) $$\begin{array}{c} {\hat{x}}_{k|k}={\hat{x}}_{k|k}^{*}+K_{k}\left(y_{k}-C_{r,k}{\hat{x}}_{k|k}^{*}\right), \end{array}$$

where ${\hat{x}}_{k-1|k-1}$ is an unbiased estimate of $x_{k-1}$ and ${\hat{x}}_{k|k-1}$ is a biased estimate. It is possible to define the innovation by

(29) $${\tilde{y}}_{k}≜y_{k}-C_{r,k}{\hat{x}}_{k|k-1}$$

and it can be concluded that

(30) $${\tilde{y}}_{k}=C_{r,k}\left(E_{r,k-1}d_{k-1}+G_{r,k-1}{\tilde{x}}_{k-1}+w_{r,k-1}\right)+v_{k}$$

with

(31) $${\tilde{x}}_{k-1}≜x_{k}-{\hat{x}}_{k|k}.$$

In the recursive filter, firstly the unknown input ${\hat{d}}_{k-1}$ is estimated from the measurement ***y**_k_* using the matrix $M_{k}$ and the biased estimate ${\hat{x}}_{k|k-1}$. Based on this estimation, the state can be estimated employing an update similar to a Kalman filter using the gain matrix $K_{k}$. The contents of this matrix can be found by analyzing the error co-variance. For evaluating the unknown input estimation, a driving maneuver is simulated. This simulated driving maneuver is driving on a circular path with a radius of 20 m. The steering angles in the front and in the back are symmetrical, but slightly different on the left and the right side in order to comply with the kinematic relationships (left modules ±1.13°; right modules ±1.16°).

**Figure 7:**
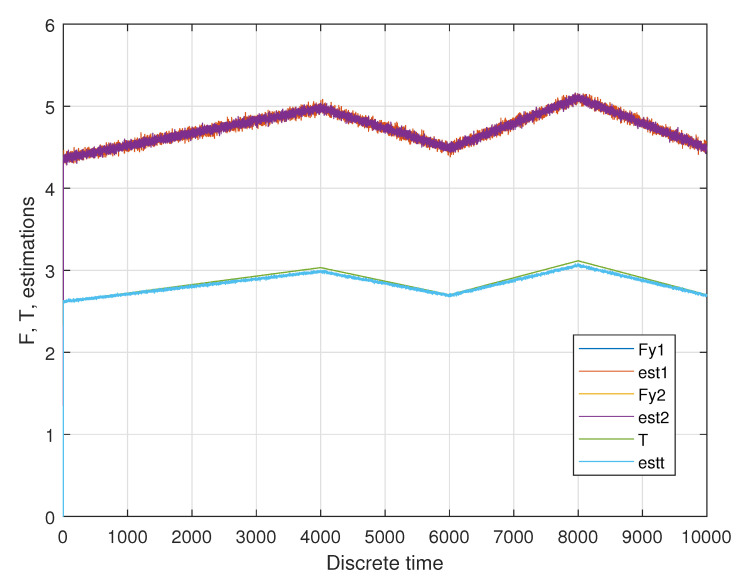
Sample estimation result for two longitudinal forces and the total torque.

In the first phase, the linear acceleration is increasing from zero to 0.05 m/s^2^; in the second phase this acceleration is going down again to 0.01 m/s^2^. In a third phase this acceleration is going up to 0.06 m/s^2^ and going down in the last phase to 0.01 m/s^2^. A sample result of the unknown input estimation for two longitudinal wheel forces of the first drive module $F_{y,1}$ and $F_{y,1}$ as well as the total torque *T* is shown in Figure 7.

It is important to note that the unit of the forces is in Newton, whereas the unit of the torque is in Newtonmeters. Based on the estimation described above, nine residual signals can be achieved, for instance a residual signal for the total torque acting on all wheels:

(32) $$\begin{array}{c} z_{T,k}=T_{k}-{\hat{T}}_{k}. \end{array}$$

Further signals concern the longitudinal forces on all eight wheels:

(33) $$\begin{array}{c} z_{y,j,k}=F_{y,j,k}-{\hat{F}}_{y,j,k}. \end{array}$$

## 6. Design of a Fuzzy Virtual Actuator

**Figure 8:**
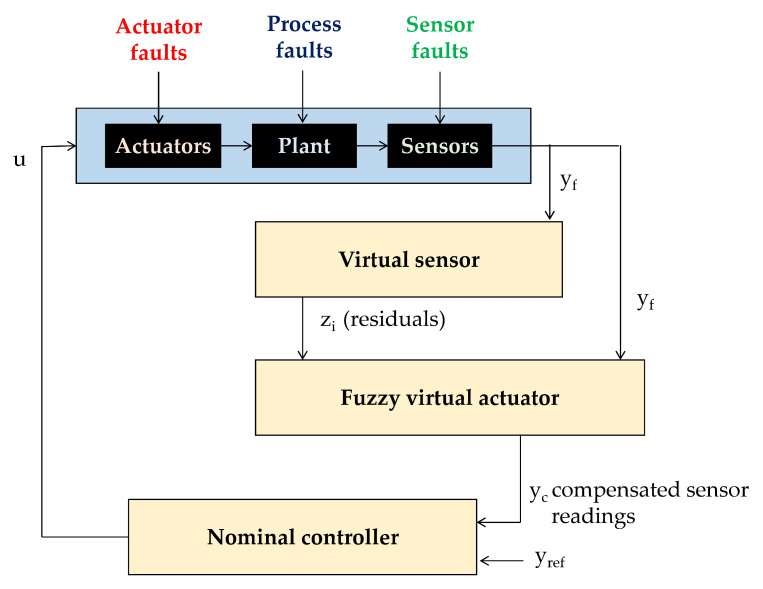
General layout of the proposed fuzzy actuator.

This section describes the structure of the fuzzy virtual actuator. This actuator analyzes the residuals that are generated using the virtual sensor (compare Section 5) and is able to determine a compensation factor, which is applied to the sensor readings in order to allow the original controller to control the AGV system. For the residual $z_{m,j}$ membership functions $\mu_{z_{m,j}}$ are derived, which allow an initial evaluation of the individual residuals. For this kind of residual evaluation, trapezoidal membership functions were found in earlier research as to be appropriate to describe the residuals in a simple and effective manner [28]. The width of the membership functions can be determined analyzing the maximum and minimum values of the residuals; those values may be found experimentally. For the accommodation of process noises, of disturbances and of mismatches between model and plant, the core is a small interval around zero, the size of which can be determined using experimental data [28]. An in-depth analysis together with design and control experts for the respective AGV made it clear that especially the combination of several residuals is beneficial for fault isolation. For instance, if one of the driving modules would be on slippery ground this will mainly be visible in the residual for the longitudinal forces of this driving module. However, also in the residual for the torque of the whole vehicle, the same influence will also be visible. This will distinguish this error from a sensor error in the respective driving module. Thus, the integrated evaluation of more than one residual will result in a higher decision certainty. This allows the application of a compensation factor to the velocity measurement of the respective driving module, which does not reflect the real vehicle speed anymore, because of the slippery surface. The general layout of the proposed fuzzy virtual actuator is depicted in Figure 8.

In our example, the expert knowledge concerning a certain fault (slippy surface under the front left driving module) was formulated with three membership functions for one of the residuals $z_{i,j}$, which are the inputs of the fuzzy system. The first input membership function $\mu_{m,j,1}$ was formulated as follows:

(34) $$\begin{array}{c} \mu_{m,j,1}=\left\{\begin{array}{cc} 1, & z_{m,j}<c_{m,j,1}\\ \frac{d_{m,j,1}-z_{m,j}}{d_{i1}-c_{m,j,1}}, & c_{m,j,1}\leq z_{i,j}\leq d_{m,j,1}\\ 0, & z_{m,j}>d_{m,j,1} \end{array}\right. \end{array}$$

In these equations, $c_{m,j,1}$ and $d_{m,j,1}$ are parameters that are determined based on expert knowledge or experimental data (all trapezoidal membership functions are defined by a lower limit *a*, an upper limit *d*, a lower support limit *b* and an upper support limit *c*, where a<b<c<d). This membership function characterises the case that a residual indicates a fault and that the value of the residual is negative, which in this case indicates that the observed longitudinal force is smaller than the force analytically expected. The next membership function $\mu_{m,j,2}$ indicates the case that the residual is close to zero, thus not indicating a fault. This membership function was formulated as follows:

(35) $$\begin{array}{c} \mu_{m,j,2}=\left\{\begin{array}{cc} 0, & \left(z_{m,j}<a_{m,j,2}\right)\;or\;\left(z_{m,j}>d_{m,j,2}\right)\\ \frac{z_{m,j}-a_{m,j,2}}{b_{m,j,2}-a_{m,j,2}}, & a_{m,j,2}\leq z_{m,j}\leq b_{m,j,2}\\ 1, & b_{m,j,2}<z_{m,j}<c_{m,j,2}\\ \frac{d_{m,j,2}-z_{m,j}}{d_{m,j,2}-c_{m,j,2}}, & c_{m,j,2}\leq z_{m,j}\leq d_{m,j,2} \end{array}\right. \end{array}$$

Also in these equations, $a_{m,j,2}$, $b_{m,j,2}$, $c_{m,j,2}$ and $d_{m,j,2}$ are parameters that are determined based on expert knowledge or experimental data. The third membership function $\mu_{m,j,3}$ can be formulated in the subsequent manner:

(36) $$\begin{array}{c} \mu_{m,j,3}=\left\{\begin{array}{cc} 0, & z_{m,j}<a_{m,j,3}\\ \frac{z_{m,j}-a_{m,j,3}}{b_{m,j,3}-a_{m,j,3}}, & a_{m,j,3}\leq z_{m,j}\leq b_{m,j,3}\\ 1, & z_{m,j}>b_{m,j,3} \end{array}\right. \end{array}$$

Also in these equations, $a_{m,j,3}$ and $b_{m,j,3}$ are parameters that are determined based on expert knowledge or experimental data. This membership characterises the case that a residual indicates a fault and that the value of the residual is positive, which in this case indicates that the observed longitudinal force is larger than the force analytically expected. It is important to note that in the given situation this case would be impossible because of the slippery surface. The presence of the residual could only be caused by another fault.

Consequently, also three membership functions are defined for the output variable, which will lead to the fault specific compensation factor Δω. The first one $\mu_{o1}$ can be formulated as follows:

(37) $$\begin{array}{c} \mu_{o1}=\left\{\begin{array}{cc} 1, & afo<c_{o1}\\ \frac{d_{o1}-afo}{d_{o1}-c_{o1}}, & c_{o1}\leq z_{m,j}\leq d_{o1}\\ 0, & afo>d_{o1} \end{array}\right. \end{array}$$

In these equations, afo stands for the aggregated fuzzy output while $c_{o1}$ and $d_{o1}$ are parameters that are determined based on expert knowledge or experimental data. A value in the range indicates that another fault (e.g., a sensor fault) has occurred. The second output membership function $\mu_{o2}$ was formulated as follows:

(38) $$\begin{array}{c} \mu_{o2}=\left\{\begin{array}{cc} 0, & afo<a_{o2}\;or\;afo>d_{o2}\\ \frac{afo-a_{o2}}{b_{o2}-a_{o2}}, & a_{o2}\leq afo\leq b_{o2}\\ 1, & b_{o2}<afo<c_{o2}\\ \frac{d_{o2}-afo}{d_{o2}-c_{o2}}, & c_{o2}\leq afo\leq d_{o2} \end{array}\right. \end{array}$$

Also in these equations, afo stands for the aggregated fuzzy output while $a_{o2}$, $b_{o2}$, $c_{o2}$ and $d_{o2}$ are parameters that are determined based on expert knowledge or experimental data. In this case, the compensation factor is zero. The third output membership function $\mu_{o3}$ can be formulated in the subsequent manner:

(39) $$\begin{array}{c} \mu_{o3}=\left\{\begin{array}{cc} 0, & afo<a_{o3}\\ \frac{afo-a_{o3}}{b_{o3}-a_{o3}}, & a_{o3}\leq afo\leq b_{o3}\\ 1, & afo>b_{o3} \end{array}\right. \end{array}$$

Also in these equations, afo stands for the aggregated fuzzy output, while $a_{o3}$ and $b_{o3}$ are parameters that are determined based on expert knowledge or experimental data. In the specific situation of the given case, it was possible to use these functions to directly determine a compensation factor. However, in many cases, additional calculations may be necessary to derive a compensation factor Δω from the direct output of the fuzzy inference system.

**Figure 9:**
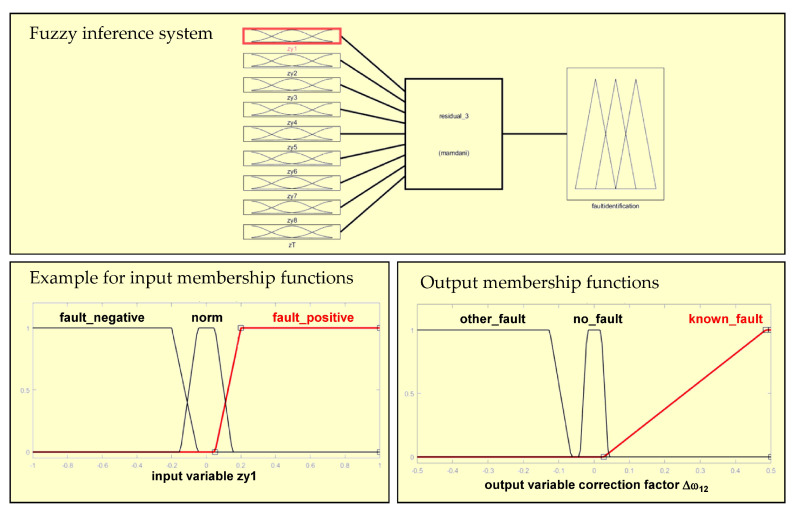
Fuzzy inference system and membership functions for residual evaluation and compensation factor generation.

Figure 9 shows the fuzzy inference system and gives an example for the membership functions of one residual (input) and the membership functions for the compensation factor.

The membership functions and rules were defined using expert knowledge. The fuzzy system allows to take information from more than one residual into consideration for a well-founded fault identification and the sizing of compensation factors for measurements.

## 7. Evaluation

**Figure 10:**
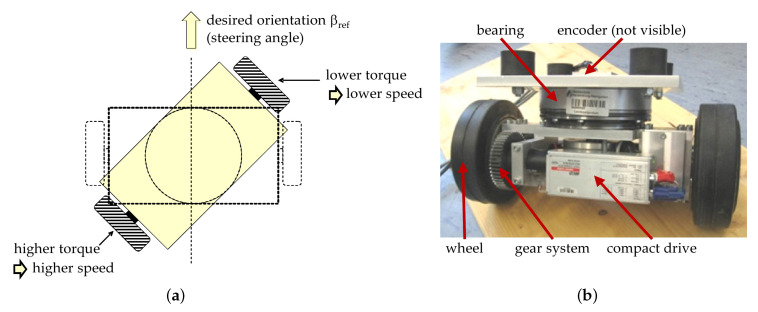
(**a**) Steering principle of the transportation platform, (**b**) driving module.

In the center of the evaluation is the transportation platform described in Section 3. As stated above, this transportation platform is employing a unique steering principle, which is based on four independent driving modules. Each driving module controls its individual steering angle through balancing the torque of two electrical motors that drive the two wheels by means of a gear system (compare Figure 10).

**Figure 11:**
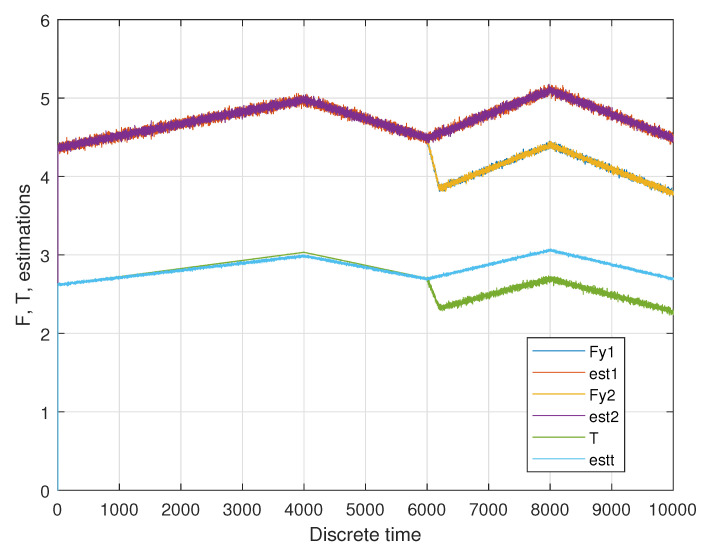
Scenario with fault at *k* = 6000.

The advantages of this arrangement are a modular design, nearly unlimited maneuvering capabilities and good preconditions for odometry. However, the detection and compensation of certain faults are mandatory, because effects such as slippery surfaces may drastically change the steering behavior and may distort the odometry readings. The transportation platform disposes of a hierarchical control system, the lower levels are the torque control of the drive motors and the steering angle control of each module (compare Figure 10a), which is realized by means of a PID (Proportional-Integral-Derivative) controller. This controller was realized within the subsystem, which consists of two electrical motors units (called compact drive—Maxon MCD EPOS—Maxon Motors, Sachseln, Switzerland—compare Figure 10b), one of them is programmable—P (Programmable) and acts as a master, the second one is not programmable and acts as a slave—S (Slave). Additionally, an absolute encoder is present, which measures the steering angle β (not visible in Figure 10, because it is inside the bearing). It was attempted to tune the controller parameters using well-known parameter tuning methods, however some parameters of the technical system needed to be estimated. Finally, a manual tuning approach was applied and resulted in a satisfactory behavior in the fault-free case. The evaluation is focusing on the next control level, where the desired steering angles for all four modules are determined and controlled. The evaluation was carried out with a focus on a certain driving maneuver within which the modules are assumed to be balanced by the dedicated PID controller (see above). As described above, the simulated driving maneuver is driving on a circular path with a radius of 20 m. The steering angles in the front and in the back are symmetrical. In the first phase up to *t* = 4000 s, the linear acceleration of the AGV is increasing; in the second phase up to *t* = 6000 s this acceleration is going down again. In a third phase up to *t* = 8000 s, this acceleration is going up again and going down in the last phase (compare Section 5). The assumed fault consists of a reduced friction on one of the modules due to a slippery surface (at *t* = 6000 s). A simulated set of forces and total torque is presented in Figure 11.

**Figure 12:**
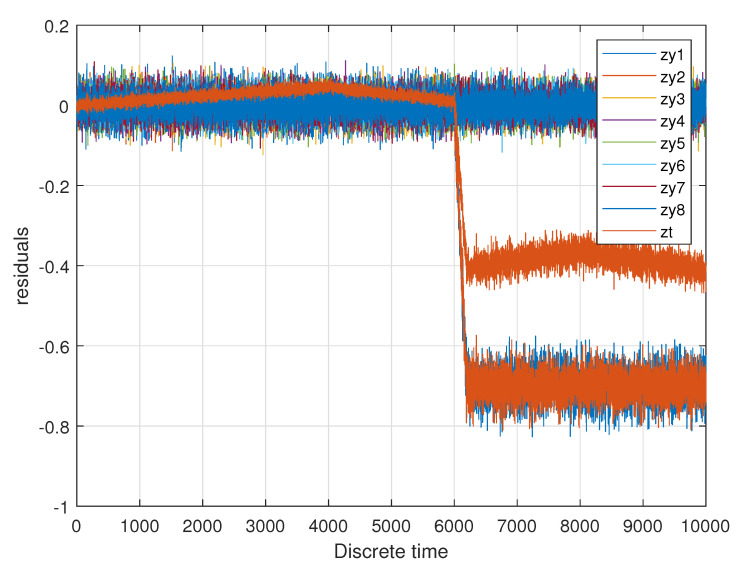
Resiudals generated by the virtual sensor.

The respective set of residuals from the virtual sensor described in Section 5 is shown in Figure 12 and is used for further deliberations.

**Figure 13:**
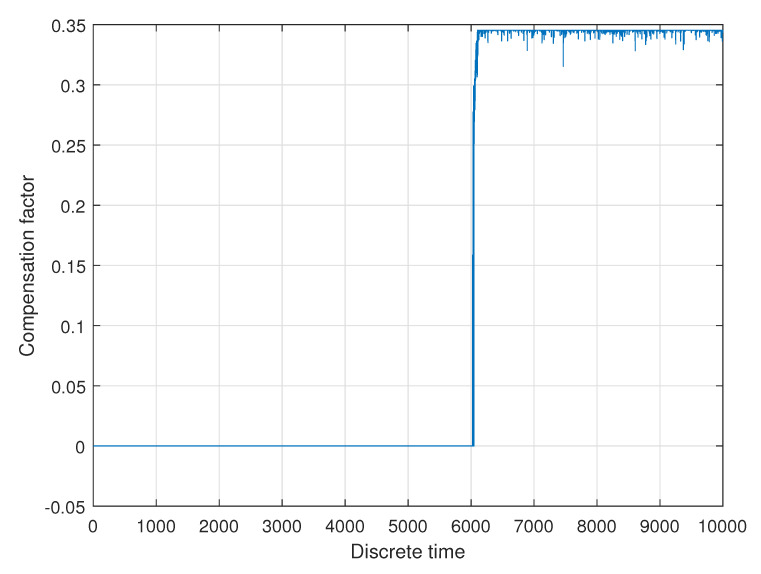
Compensation factor $\omega_{12}$ generated by the virtual actuator.

The residuals were used as an input for the realized fuzzy logic system, which initially has individual membership functions for each residual. The rules, which are based on expert knowledge, combine the information of all residuals in order to allow a decision as to whether a certain fault is present. Due to the special nature of fuzzy logic, it is also possible to assess the size of the fault and to determine a compensation factor for one of the measurements (compare Figure 13).

When analyzing the information shown in Figure 13, firstly it becomes obvious that, as soon as the fault occurs, the compensation factor is set to a sensible numerical value (at t=6000 s). The acceleration changes at t=4000 s and t=8000 s do not change the compensation factor, which is sensible. The disturbances present in the residuals are reduced. The given fault—one of the driving modules on slippery ground was visible in the residual for the two longitudinal forces of this driving module and also in the residual for the torque of the whole vehicle. This redundant information can be used as the virtual actuator to distinguish this fault from, for example, a sensor malfunction in the respective driving module. Further investigations showed that another scenario, within which only one residual was changing, lead to the conclusion by the fuzzy inference systems that the specific fault is not present and the compensation factor remained close to zero. The feasibility of the presented system could be demonstrated, because when the fault appears, the compensation factor is set to a sensible numerical value, which enables an unchanged controller to control the vehicle. This controller guarantees the stability, satisfactory and efficient performance, as well as robust operation in nominal conditions (compare [2]). The fuzzy virtual actuator achieves a certain decision reliability by taking into account more than one residual and expert knowledge or experimental data.

## 8. Conclusions and Outlook

This paper describes the design of a fuzzy virtual actuator for an automated guided vehicle. The central focus is the development of a system structure and algorithms for a control and diagnosis subsystem for an AGV, which realizes a fuzzy virtual actuator. This actuator is employing the information of residuals created with an appropriate analytical model of the AGV and is combining this information with expert knowledge. The novel concept exhibits the advantageous quality of being able to sensibly combine information from more than one residual for enlarging the decision certainty in fault detection and isolation. The ultimate aim is an increase of the fault-tolerance of the respective AGV. Today, several forms of virtual actuators were proposed in the literature, however, until now, only one class of fuzzy virtual actuators was mentioned in the literature; this mentioned class is focusing on the representation of a nonlinear system by means of the Takagi–Sugeno fuzzy model. In this paper, a completely new class of fuzzy virtual actuators is proposed. The central focus is on the application of fuzzy rules, which represent the expert knowledge and/or experimental results. The main advantage of the underlying concept is the possibility to combine the information from several residuals in order to enhance the decision certainty in fault detection and isolation. The second advantage lies in the possibility to derive a numerical value for compensation factors, which is based on an analysis of more than one residual and expert knowledge. Together with a virtual sensor design based on an unknown input estimator, a novel and powerful FDI scheme could be generated and demonstrated. The unique contribution is the combination of a virtual sensor based analytical redundancy, which leads to meaningful residuals, with a fuzzy inference system that combines the information of more than one residual and incorporates expert knowledge. The demonstration of the feasibility concentrated on a certain AGV, but an application for controlling AGVs based on different kinematic systems can be presumed. Due to modular design of the approach, an expansion to other control applications is probably possible. In the literature, several alternative approaches that can create residuals are proposed and could also be integrated in the control and diagnosis subsystem. An interesting application field is the active vibration control of mechanical systems and especially virtual dynamic vibration absorbers. These virtual absorbers employ, for example, electrical motors and a control system in order to realize, for example, multimode vibration absorbers. It might be possible to include a fuzzy virtual actuator in this control system either for combing sensory information or for fault identification and compensation. Further research in this direction would be highly desirable. Research that investigates the application of fuzzy virtual actuators in distributed and wireless environments will also be fruitful because the possibility to combine information from several residuals and to include other kinds of information such as expert knowledge might also be advantageous in the scope of IoT. So far, the feasibility of the presented approach was demonstrated in an initial evaluation. Extensive experimental investigations are planned in order to investigate the strengths and weaknesses of the approach. Additionally, the investigation of stability will be an important future research topic. A detailed comparison with other forms of virtual actuators is also a topic for future scientific activities. Future research work will also focus on an expansion to the control of other systems and a systematic formulation scheme for membership functions and rules.

## Figures and Tables

**Table 1:** Notation.

COG	center of gravity
i=1,2,3,4	number of drive modules
j=1,⋯,8	number of wheels
m=x,y,z	directions
*b*	distance between modules and COG (*y*-direction)
*a*	distance between modules and COG (*x*-direction)
$d_{j}$	distance between wheel contact point of the *j*-th wheel and the ICR
$L_{m}$	drive module gauge
α	vehicle angle
$\dot{\alpha }$	vehicle angular velocity (yaw rate)
$\ddot{\alpha }$	vehicle angular acceleration
$x_{w},y_{w}$	position of the vehicle in the world coordinate system
$x_{c},y_{c}$	position of the vehicle in the center coordinate system
$\dot{x_{c}}$	lateral velocity of the vehicle
$\dot{y_{c}}$	longitudinal velocity of the vehicle
$\ddot{x_{c}}$	lateral acceleration of the vehicle
$\ddot{y_{c}}$	longitudinal acceleration of the vehicle
*m*	mass
$\beta_{i}$	steering angle of the *i*-th drive module
$\dot{\beta_{i}}$	angular velocity of the *i*-th drive module
$\ddot{\beta_{i}}$	angular acceleration of the *i*-th drive module
$\gamma_{j}$	angle of the *j*-th wheel
$\dot{\gamma_{j}}$	angular velocity of the *j*-th wheel
$\ddot{\gamma_{j}}$	angular acceleration of the *j*-th wheel
$F_{xc}$	sum of forces causing lateral motion
$F_{yc}$	sum of forces causing longitudinal motion
$F_{y,j}$	longitudinal force on the *j*-th wheel
$F_{x,j}$	total lateral force on the *j*-th wheel
*T*	total torque acting on all wheels
$p_{j}$	torque distribution coefficient
κ	coefficient summarizing additional inertia in the drivetrain
$\mu_{r}$	rolling friction coefficient
$I_{w}$	wheel moment of inertia
$I_{m}$	drive module moment of inertia
$I_{z}$	AGV moment of inertia around *z*-axis
$R_{e}$	wheel effective radius

**Table 2:** Essential parameters.

Variable	Unit	Value
*m*	kg	89
$R_{e}$	m	0.075
*a*	m	0.196
*b*	m	0.399
κ		1.1
$\mu_{r}$		0.04
$I_{z}$	$\mathrm{kg}/m^{2}$	578.18
$I_{m}$	$\mathrm{kg}/m^{2}$	4.15
$I_{w}$	$\mathrm{kg}/m^{3}$	0.00871513

## Abbreviations

The following abbreviations are used in this manuscript:

AFAC	Actuator failure avoidance online charging schemes
AGV	Automated guided vehicle
A-NSGA	Non-dominated sorting based genetic algorithm
CPN	Colored Petri nets
FDI	Fault detection and identification
FRVPN	Fuzzy reasoning and verification Petri net
FTC	Fault-tolerant control
IoT	Internet of Things
LPV	Linear parameter varying
PID	Proportional-Integral-Derivative
RUL	Remaining useful life
SLAM	Simultaneous localization and mapping
TS	Takagi–Sugeno

## Appendix A

The system matrices in the state space model (16) described in Section 4 are:

(A1) $$\begin{array}{c} G=\left[\begin{array}{ccccccccccccc} p_{a} & 0 & 0 & 0 & 0 & 0 & 0 & 0 & 0 & 0 & 0 & 0 & 0\\ 0 & 1 & 0 & 0 & 0 & 0 & 0 & 0 & 0 & 0 & 0 & 0 & 0\\ 0 & 0 & 1 & 0 & 0 & 0 & 0 & 0 & 0 & 0 & 0 & 0 & 0\\ 0 & 0 & 0 & 1 & 0 & 0 & 0 & 0 & 0 & 0 & 0 & 0 & 0\\ 0 & 0 & 0 & 0 & 1 & 0 & 0 & 0 & 0 & 0 & 0 & 0 & 0\\ 0 & 0 & 0 & 0 & 0 & 1 & 0 & 0 & 0 & 0 & 0 & 0 & 0\\ 0 & 0 & 0 & 0 & 0 & 0 & 1 & 0 & 0 & 0 & 0 & 0 & 0\\ 0 & 0 & 0 & 0 & 0 & 0 & 0 & 1 & 0 & 0 & 0 & 0 & 0\\ 0 & 0 & 0 & 0 & 0 & 0 & 0 & 0 & 1 & 0 & 0 & 0 & 0\\ 0 & 0 & 0 & 0 & 0 & 0 & 0 & 0 & 0 & 1 & 0 & 0 & 0\\ 0 & 0 & 0 & 0 & 0 & 0 & 0 & 0 & 0 & 0 & 1 & 0 & 0\\ 0 & 0 & 0 & 0 & 0 & 0 & 0 & 0 & 0 & 0 & 0 & 1 & 0\\ 0 & 0 & 0 & 0 & 0 & 0 & 0 & 0 & 0 & 0 & 0 & 0 & 1 \end{array}\right] \end{array}$$

(A2) $$\begin{array}{c} B=\left[\begin{array}{cc} p_{x} & p_{y}\\ 0 & 0\\ 0 & 0\\ 0 & 0\\ 0 & 0\\ 0 & 0\\ 0 & 0\\ 0 & 0\\ 0 & 0\\ 0 & 0\\ 0 & 0\\ 0 & 0\\ 0 & 0 \end{array}\right] \end{array}$$

(A3) E=−py1−py2−py3−py4−py5−py6−py7−py80−Lm2·ImLm2·Im000000000−Lm2·ImLm2·Im000000000−Lm2·ImLm2·Im000000000−Lm2·ImLm2·Im0−ReIw0000000p1Iw0−ReIw000000p2Iw00−ReIw00000p3Iw000−ReIw0000p4Iw0000−ReIw000p5Iw00000−ReIw00p6Iw000000−ReIw0p7Iw0000000−ReIwp8Iw

where

$$p_{a}=\frac{p_{alpha}}{I}$$

$$p_{alpha}=(2\cdot sin\left(-\beta_{3}+\beta_{1}\right)+2\cdot sin\left(\beta_{1}-\beta_{4}\right)+2\cdot sin\left(-\beta_{3}+\beta_{2}\right)+2\cdot sin\left(-\beta_{4}+\beta_{2}\right)$$

$$\begin{array}{c} p_{y1}=(-4\cdot sin\left(\beta_{4}\right)\cdot a-2\cdot a\cdot sin\left(\beta_{4}-\beta_{2}+\beta_{1}\right)+2\cdot a\cdot sin\left(-\beta_{4}+\beta_{2}+\beta_{1}\right)+\\ 2\cdot a\cdot sin\left(-\beta_{3}+\beta_{2}+\beta_{1}\right)-2\cdot a\cdot sin\left(-\beta_{3}-\beta_{2}+\beta_{1}\right)-2\cdot b\cdot cos\left(-\beta_{3}-\beta_{2}+\beta_{1}\right)-\\ 2\cdot b\cdot cos\left(-\beta_{3}+\beta_{2}+\beta_{1}\right)-2\cdot b\cdot cos\left(-\beta_{4}+\beta_{2}+\beta_{1}\right)-2\cdot b\cdot cos\left(-\beta_{4}-\beta_{2}+\beta_{1}\right) \end{array}$$

$$\begin{array}{c} p_{y2}=(-4\cdot sin\left(\beta_{4}\right)\cdot a-2\cdot a\cdot sin\left(\beta_{4}-\beta_{2}+\beta_{1}\right)+2\cdot a\cdot sin\left(-\beta_{4}+\beta_{2}+\beta_{1}\right)+\\ 2\cdot a\cdot sin\left(-\beta_{3}+\beta_{2}+\beta_{1}\right)-2\cdot a\cdot sin\left(-\beta_{3}-\beta_{2}+\beta_{1}\right)-2\cdot b\cdot cos\left(-\beta_{3}-\beta_{2}+\beta_{1}\right)-\\ 2\cdot b\cdot cos\left(-\beta_{3}+\beta_{2}+\beta_{1}\right)-2\cdot b\cdot cos\left(-\beta_{4}+\beta_{2}+\beta_{1}\right)-2\cdot b\cdot cos\left(-\beta_{4}-\beta_{2}+\beta_{1}\right) \end{array}$$

$$\begin{array}{c} p_{y3}=(-sin\left(\beta_{3}\right)\cdot a-a\cdot sin\left(-\beta_{4}+2\cdot \beta_{2}\right)+sin\left(\beta_{4}\right)\cdot a-2\cdot a\cdot sin\left(-\beta_{3}+2\cdot \beta_{2}\right)+\\ a\cdot sin\left(\beta_{3}+2\cdot \beta_{2}\right)+a\cdot sin\left(\beta_{4}-\beta_{2}+\beta_{1}\right)-2\cdot a\cdot sin\left(-\beta_{4}-\beta_{2}+\beta_{1}\right)-a\cdot sin(\beta_{4}+\\ \beta_{2}+\beta_{1})-a\cdot sin\left(-\beta_{3}+\beta_{2}+\beta_{1}\right)-a\cdot sin\left(-\beta_{3}-\beta_{2}+\beta_{1}\right)+b\cdot cos\left(-\beta_{4}+2\cdot \beta_{2}\right)-\\ b\cdot cos\left(\beta_{4}+2\cdot \beta_{2}\right)+b\cdot cos\left(-\beta_{3}+2\cdot \beta_{2}\right)-b\cdot cos\left(\beta_{3}+2\cdot \beta_{2}\right) \end{array}$$

$$\begin{array}{c} p_{y4}=(-a\cdot sin\left(\beta_{4}-\beta_{2}+\beta_{1}\right)-3\cdot b\cdot cos\left(\beta_{3}+2\cdot \beta_{2}\right)-3\cdot a\cdot sin\left(\beta_{4}+\beta_{2}+\beta_{1}\right)-\\ sin\left(\beta_{4}\right)\cdot a+3\cdot a\cdot sin\left(\beta_{3}+2\cdot \beta_{2}\right)-4\cdot cos\left(\beta_{4}\right)\cdot b-2\cdot a\cdot sin\left(-\beta_{3}+2\cdot \beta_{2}\right)+\\ sin\left(\beta_{3}\right)\cdot a-3\cdot a\cdot sin\left(-\beta_{3}-\beta_{2}+\beta_{1}\right)-4\cdot cos\left(\beta_{3}\right)\cdot b-b\cdot cos(-\beta_{4}+\\ 2\cdot \beta_{2})-3\cdot a\cdot sin\left(-\beta_{3}+\beta_{2}+\beta_{1}\right)-b\cdot cos\left(-\beta_{3}+2\cdot \beta_{2}\right)+a\cdot sin\left(-\beta_{4}+2\cdot \beta_{2}\right)-\\ 2\cdot a\cdot sin\left(-\beta_{4}-\beta_{2}+\beta_{1}\right)-3\cdot b\cdot cos\left(\beta_{4}+2\cdot \beta_{2}\right) \end{array}$$

$$\begin{array}{c} p_{y5}=(-4\cdot cos\left(\beta_{2}\right)\cdot b+4\cdot sin\left(\beta_{2}\right)\cdot a+2\cdot a\cdot sin\left(-\beta_{4}-\beta_{3}+\beta_{2}\right)+2\cdot a\cdot sin(-\beta_{4}+\\ \beta_{3}+\beta_{2})-2\cdot a\cdot sin\left(\beta_{1}-\beta_{3}+\beta_{4}\right)+2\cdot a\cdot sin\left(\beta_{1}-\beta_{3}-\beta_{4}\right)-2\cdot b\cdot cos(-\beta_{4}+\\ \beta_{3}+\beta_{2})-2\cdot b\cdot cos\left(\beta_{4}-\beta_{3}+\beta_{2}\right) \end{array}$$

$$\begin{array}{c} p_{y6}=(-4\cdot cos\left(\beta_{2}\right)\cdot b+4\cdot sin\left(\beta_{2}\right)\cdot a+2\cdot a\cdot sin\left(-\beta_{4}-\beta_{3}+\beta_{2}\right)+2\cdot a\cdot sin(-\beta_{4}+\\ \beta_{3}+\beta_{2})-2\cdot a\cdot sin\left(\beta_{1}-\beta_{3}+\beta_{4}\right)+2\cdot a\cdot sin\left(\beta_{1}-\beta_{3}-\beta_{4}\right)-2\cdot b\cdot cos(-\beta_{4}+\beta_{3}+\\ \beta_{2})-2\cdot b\cdot cos\left(\beta_{4}-\beta_{3}+\beta_{2}\right) \end{array}$$

$$\begin{array}{c} p_{y7}=(-4\cdot cos\left(\beta_{2}\right)\cdot b-4\cdot sin\left(\beta_{1}\right)\cdot a-2\cdot a\cdot sin\left(-\beta_{4}-\beta_{3}+\beta_{2}\right)+2\cdot a\cdot sin(-\beta_{4}+\\ \beta_{3}+\beta_{2})-2\cdot a\cdot sin\left(\beta_{1}-\beta_{3}+\beta_{4}\right)-2\cdot a\cdot sin\left(\beta_{1}-\beta_{3}-\beta_{4}\right)-2\cdot b\cdot cos(-\beta_{4}+\beta_{3}+\\ \beta_{2})-2\cdot b\cdot cos\left(\beta_{4}-\beta_{3}+\beta_{2}\right) \end{array}$$

$$\begin{array}{c} p_{y8}=(-b\cdot cos\left(\beta_{1}-\beta_{3}-\beta_{4}\right)-b\cdot cos\left(\beta_{4}-\beta_{3}+\beta_{2}\right)+cos\left(\beta_{4}\right)\cdot b+b\cdot cos(\beta_{4}-\beta_{2}+\\ \beta_{1})-b\cdot cos\left(-2\cdot \beta_{4}+\beta_{2}\right)-2\cdot b\cdot cos\left(-\beta_{4}+\beta_{3}+\beta_{2}\right)-b\cdot cos\left(-\beta_{4}-\beta_{3}+\beta_{2}\right)-\\ b\cdot cos\left(2\cdot \beta_{1}-\beta_{4}\right)-2\cdot a\cdot sin\left(\beta_{1}-\beta_{3}+\beta_{4}\right)+b\cdot cos\left(\beta_{1}-\beta_{3}+\beta_{4}\right)+\\ 2\cdot a\cdot sin\left(-\beta_{4}+\beta_{3}+\beta_{2}\right)-b\cdot cos\left(-\beta_{3}+\beta_{2}+\beta_{1}\right)+b\cdot cos\left(\beta_{3}-\beta_{2}+\beta_{1}\right)-\\ 2\cdot a\cdot sin\left(-\beta_{4}-\beta_{3}+\beta_{2}\right)-b\cdot cos\left(-2\cdot \beta_{4}+\beta_{1}\right)+cos\left(\beta_{3}\right)\cdot b-4\cdot sin\left(\beta_{1}\right)\cdot a-\\ 2\cdot a\cdot sin\left(\beta_{1}-\beta_{3}-\beta_{4}\right)-3\cdot cos\left(\beta_{2}\right)\cdot b+cos\left(\beta_{1}\right)\cdot b-b\cdot cos\left(2\cdot \beta_{1}-\beta_{3}\right)-\\ b\cdot cos(-\beta_{4}+\beta_{2}+\beta_{1}) \end{array}$$

$$\begin{array}{c} p_{x}=(2\cdot cos\left(-\beta_{3}+\beta_{2}\right)\cdot a\cdot m-2\cdot cos\left(\beta_{3}+\beta_{2}\right)\cdot a\cdot m-2\cdot cos\left(\beta_{1}-\beta_{4}\right)\cdot a\cdot m+\\ 2\cdot cos\left(\beta_{1}+\beta_{4}\right)\cdot a\cdot m-2\cdot sin\left(\beta_{1}-\beta_{4}\right)\cdot b\cdot m-2\cdot sin\left(-\beta_{3}+\beta_{1}\right)\cdot b\cdot m-\\ 2\cdot sin\left(\beta_{4}+\beta_{2}\right)\cdot b\cdot m-2\cdot sin\left(\beta_{3}+\beta_{2}\right)\cdot b\cdot m) \end{array}$$

$$\begin{array}{c} p_{y}=(2\cdot cos\left(-\beta_{4}+\beta_{2}\right)\cdot b\cdot m+2\cdot cos\left(\beta_{4}+\beta_{2}\right)\cdot b\cdot m+2\cdot cos\left(-\beta_{3}+\beta_{2}\right)\cdot b\cdot m+\\ 2\cdot cos\left(\beta_{3}+\beta_{2}\right)\cdot b\cdot m+2\cdot sin\left(\beta_{1}+\beta_{4}\right)\cdot a\cdot m+2\cdot sin\left(-\beta_{3}+\beta_{1}\right)\cdot \\ a\cdot m-2\cdot sin\left(-\beta_{4}+\beta_{2}\right)\cdot a\cdot m-2\cdot sin\left(\beta_{3}+\beta_{2}\right)\cdot a\cdot m) \end{array}$$
